# Supplementary material for: In vivo CRISPR screening identifies SAGA complex members as key regulators of hematopoiesis
Source: Nat Commun. 2026 Jan 23;17:1756. doi: 10.1038/s41467-026-68465-6 (PMC12914052; doi:10.1038/s41467-026-68465-6)
Supplement: Supplementary file 1 — Supplementary Information [file 41467_2026_68465_MOESM1_ESM.pdf]

In vivo CRISPR screening identifies SAGA complex members as key regulators of hematopoiesis: Supplementary Information

Supplementary Table 1: List of materials used in this study

**REAGENT or RESOURCE**	**SOURCE**	**IDENTIFIER**
Antibodies		
Biotin anti-mouse CD4	Biolegend	100508
Biotin anti-mouse CD8a	Biolegend	100704
Biotin anti-mouse TER-119/Erythroid Cells	Biolegend	116204
Biotin anti-mouse CD127 (IL-7Rα)	Biolegend	135006
Biotin anti-mouse Ly-6G/Ly-6C (Gr-1)	Biolegend	108404
Biotin anti-mouse/human CD45R/B220	Biolegend	103204
APC/Cyanine7 Streptavidin	Biolegend	405208
FITC anti-mouse CD4	Biolegend	100510
FITC anti-mouse CD8a	Biolegend	100706
FITC anti-mouse CD127 (IL-7Rα)	Biolegend	135008
FITC anti-mouse Ter119	Biolegend	116206
FITC anti-mouse/human CD45R/B220	Biolegend	103206
FITC anti-mouse Ly-6G/Ly-6C (Gr-1)	Biolegend	108406
Brilliant Violet 421 anti-mouse CD117 (c-Kit)	Biolegend	105828
PE anti-mouse Ly-6A/E (Sca-1)	Biolegend	108108
PE/Cyanine7 anti-mouse CD150 (SLAM)	Biolegend	115914
APC anti-mouse CD201 (EPCR)	Thermo Fisher	17-2012-82
PE/Cyanine7 anti-mouse CD45.1	Biolegend	110730
eFluor 450 anti-mouse CD45.2	Thermo Fisher	48-0454-82
FITC anti-mouse CD34	Thermo Fisher	11-0341-85
Brilliant Violet 711 anti-mouse CD16/32 (Fcγ R III/II)	Biolegend	101337
APC anti-mouse CD117 (c-Kit)	Biolegend	105812
Alexa Fluor 700 anti-mouse H-2Kb (MHC class I)	Biolegend	116522
BUV395 Mouse Anti-Mouse CD45.2	BD Biosciences	564616
Brilliant Violet 421 anti-mouse CD48	Biolegend	103428
Brilliant Violet 785 anti-mouse CD150 (SLAM)	Biolegend	115937
APC anti-mouse CD4	Biolegend	100516
APC anti-mouse CD8a	Biolegend	100712
FITC anti-mouse CD41	Biolegend	133903
APC/Cy7 anti-mouse CD41	Biolegend	133927
PE anti-mouse/human CD11b	Biolegend	101208
PE anti-mouse Ly-6G/Ly-6C (Gr-1)	Biolegend	108408
APC/Cyanine7 anti-mouse/human CD45R/B220	Biolegend	103224
Alexa Fluor 700 anti-mouse CD34	Thermo Fisher	56-0341-82
APC anti-mouse Ter119	Biolegend	116212
APC anti-mouse/human CD11b	Biolegend	101212
APC anti-mouse Ly-6G/Ly-6C (Gr-1)	Biolegend	108412
APC anti-mouse/human CD45R	Biolegend	103212
Microbead-conjugated anti-APC	Miltenyi	130-090-855

Microbead-conjugated anti-mouse CD117 (c-Kit)	Miltenyi	130-097-146
APC anti-human CD41	Biolegend	303710
APC/Cy7 anti-human CD34	Biolegend	343514
PE anti-human CD201	Biolegend	351904
BV785 anti-human CD45RA	Biolegend	304140
Anti-mouse TADA2B	St. John's Laboratory	STJ194361-200
Anti-mouse TAF5L	Proteintech	19274-AP
Anti-mouse TADA1	Proteintech	20337-1-AP
Anti-mouse RUNX1	Cell Signaling	8529s
Anti-mouse histone H3	Abcam	ab1791
Anti-mouse GAPDH	Bethyl Laboratories	A300-641A
PE Anti-acetyl histone H3 Lys9	Cell Signaling	28036
PE Anti-ubiquityl-histone H2B Lys120	Cell Signaling	86653
Anti-acetyl histone H3 Lys9	Merck Millipore	07-352
Anti-ubiquityl histone H2B Lys120	Cell Signaling	5546
Peroxidase conjugated goat anti-rabbit IgG	Sigma Aldrich	A6667
**Biological samples**		
Umbilical cord blood CD34^{+} cells	NHSBT	NCB010
**Chemicals, peptides, and recombinant proteins**		
Propidium Iodide Solution	Sigma Aldrich	P4864
Polyvinyl alcohol	Sigma Aldrich	P8136
Soluplus	BASF	50539897
Recombinant Cas9	IDT	1081059
Recombinant mouse thrombopoietin (THPO)	Peprotech	AF-315-14
Recombinant mouse stem cell factor (SCF)	Peprotech	AF-250-03
Recombinant human interleukin-3 (IL-3)	Peprotech	200-03
Recombinant human FLT3 ligand (FLT3L)	Peprotech	AF-300-19
Recombinant mouse interferon alpha	Biolegend	752804
Mitotracker Green FM	Thermo Fisher	M46750
Mitotracker Orange CMTMRos	Thermo Fisher	M7510
GSK-699	InvivoChem	V41244
IACS-010759	Selleckchem	S8731
740 Y-P	MedChemExpress	HY-P0175
UM729	Stem Cell Technologies	72332
Butzyamide	CellAid	N/A
ITSX	Thermo Fisher	51500056
PSG	Thermo Fisher	10378016
Ham's F12 media	Thermo Fisher	11765054
IMDM	Thermo Fisher	12440061
RPMI1640	Thermo Fisher	21875034
Defined FBS	Corning	35-010-CV
BD Transcription Factor Buffer set	BD Pharmingen	562574
**Critical commercial assays**		

Chromium GEM single cell 3' kit v3	10X Genomics	PN-1000268
P3 Primary Cell Nucleofector Kit	Lonza	V4SP-3096
Precision Plus Protein Kaleidoscope kit	Biorad	1610375
Quick-RNA microprep kit	Zymo Research	R1055
SSIV Reverse Transcriptase kit	Thermo Fisher	18090050
**Experimental models: Cell lines**		
NIH/3T3 cell line	ATCC	CRL-1658
MDS-L cell line	Kida et al. 2018	
**Organisms/strains**		
B6J.129(Cg)-Gt(ROSA)26Sortm1.1(CAG-cas9*,-EGFP)Fezh/J	JAX	026179
PepboyJ	JAX	002014
C57BL/6	JAX	000664
C6.129S2-Ifnar1tm1Agt	MMRRC	032045
NOD.Cg-Prkdcscid Il2rgtm1SugTg(SV40/HTLV-IL3,CSF2)10-7Jic/JicTac	Taconics Biosciences	13395-F
**Oligonucleotides**		
Rosa26 sgRNA - ACUCCAGUCUUUCUAGAAGA	Thermo Fisher, Synthego	
Rosa26 fwd primer - ggctgttttggaggcaggaag	IDT	
Rosa26 rev primer - ccgaggcggatcacaagcaata	IDT	
Tada2b sgRNA 1 - CGGCGGACGAUUCACACUCU	Thermo Fisher, Synthego	
Tada2b sgRNA 2 - UGGGGUCCUGAGGCGGAGGG	Thermo Fisher, Synthego	
Tada2b sgRNA 3 - GCCGAAGCCGAACUGCUCGA	Thermo Fisher, Synthego	
Tada2b fwd primer - GGGATCTAGCTTGCTGCCAT	IDT	
Tada2b rev primer - GTACTGCTTGGCCGAGGTG	IDT	
Tada2b sequencing primer - ATAAGCCTGGCAACGCACAG	IDT	
Taf5l sgRNA 1 - UGGCACAACCAGAUUCUGAU	Thermo Fisher, Synthego	
Taf5l sgRNA 2 - UGGGGCUGCAGACACCGCAU	Thermo Fisher, Synthego	
Taf5l sgRNA 3 - AAACUGCACUUCAUACUGCU	Thermo Fisher, Synthego	
Taf5l fwd primer - ATTACGGTGCCAAAAGCCAC	IDT	
Taf5l rev primer - TCTCAGGAATATTGCGTGCT	IDT	
Taf5l sequencing primer - ATTGCGTGCTTCTCTTCTCT	IDT	
Taf6l sgRNA 1 - UUUUCCACUUGCAGUUCCAC	Thermo Fisher, Synthego	

Taf6l sgRNA 2 - CUCUCGUUCUGACAUGGCCC	Thermo Fisher, Synthego	
Taf6l sgRNA 3 - GGAGGUUUGUGGAGAUCCCU	Thermo Fisher, Synthego	
Taf6l fwd primer - TGCCCAGCGAGTGTTTTCTT	IDT	
Taf6l rev primer - CTTGGACTAGGGTCGTGCTG	IDT	
Taf6l sequencing primer - TTCTAGACACCTGACTGGCT	IDT	
Tada1 sgRNA 1 - ACUGGGCCAACCUGAAGUUG	Thermo Fisher	
Tada1 sgRNA2 - GGCGACCUUUGUGAGCGAGC	Thermo Fisher	
Tada1 sgRNA 3 - UCUGCUUGAACCACAACUUC	Thermo Fisher	
Tada1 fwd primer (for sgRNAs 1 and 3) - GCTCATCTGAACGGAAGCGT	IDT	
Tada1 rev primer (for sgRNAs 1 and 3) - CACCCCCTTACCTGGTGTAG	IDT	
Tada1 sequencing primer (for sgRNAs 1 and 3) - ACCTCGTGGTCTCCTCCTAA	IDT	
Tada1 fwd primer (for sgRNA 2) - GCCGCGTTGATCTTTCGGTTGC	IDT	
Tada1 rev primer (for sgRNA 2) - ACGCCCAGGAGAAAGTCCTCCAG	IDT	
Runx1 sgRNA 1 - GCGCCTCGCTCATCTTGCCG	Thermo Fisher	
Runx1 sgRNA 2 - CGGTGCGCACTAGCTCGCCA	Thermo Fisher	
Runx1 fwd primer - CAATACTTGCGGCTTCTGGC	IDT	
Runx 1 rev primer - AATCGGCTTGTTGTGATGCG	IDT	
Runx 1 sequencing primer - TTCTACTACAGGCAGCACCC	IDT	
Mouse Ifit Taqman primers/probe	Thermo Fisher	Mm00515153_m1
Mouse Gapdh Taqman primers/probe	Thermo Fisher	Mm99999915_g1
**Recombinant DNA**		
pLL3.7-shControl	Arede et al. 2022	
pLL3.7-shTADA2B	Arede et al. 2022	
pcDNA3.1-Ubc-GFP	This paper	
pcDNA3.1-Ubc-Tada2b-T2A-GFP	This paper	
pCMV-VSV-G-RSV-Rev	Addgene	8454
pMDLg/pRRE	Addgene	12251
pMD2.G	Addgene	12259
psPAX2	Addgene	12260

Bassik mouse CRISPR knockout library	Addgene	1000000121-1000000130
pMCB306	Addgene	89360
pLentiCas9-Blast	Addgene	52962
**Software**		
FACSDiva	BD	Version 8
FlowJo	BD	Version 10.8.0
GraphPad Prism	GraphPad Software LLC	Version 10.1.1
CasTLE	Morgens et al. 2016	Version 1.0
DESeq2	Love et al. 2014	Version 1.42.1
Seurat	Butler et al. 2018	Version 2.0

Supplementary Table 2: Haematopoietic stem and progenitor cell immunophenotypes

**Cell type**	**Immunophenotype**
LT-HSC	CD150$^+$CD48$^-$CD34$^{-/lo}$c-Kit$^+$Sca-1$^+$Lineage$^-$
ST-HSC/LT-HSC	CD34$^{-/lo}$c$^-$Kit$^+$Sca-1$^+$Lineage$^-$
pHSC/MPP	c-Kit$^+$Sca-1$^+$Lineage$^-$
CMP	CD16/32$^-$CD34$^+$Sca-1$^-$Lineage$^-$
GMP	CD16/32+CD34$^+$Sca-1$^-$Lineage$^-$
MEP	CD16/32$^-$CD34$^-$Sca-1$^-$Lineage$^-$

Supplementary Figures and Legends:

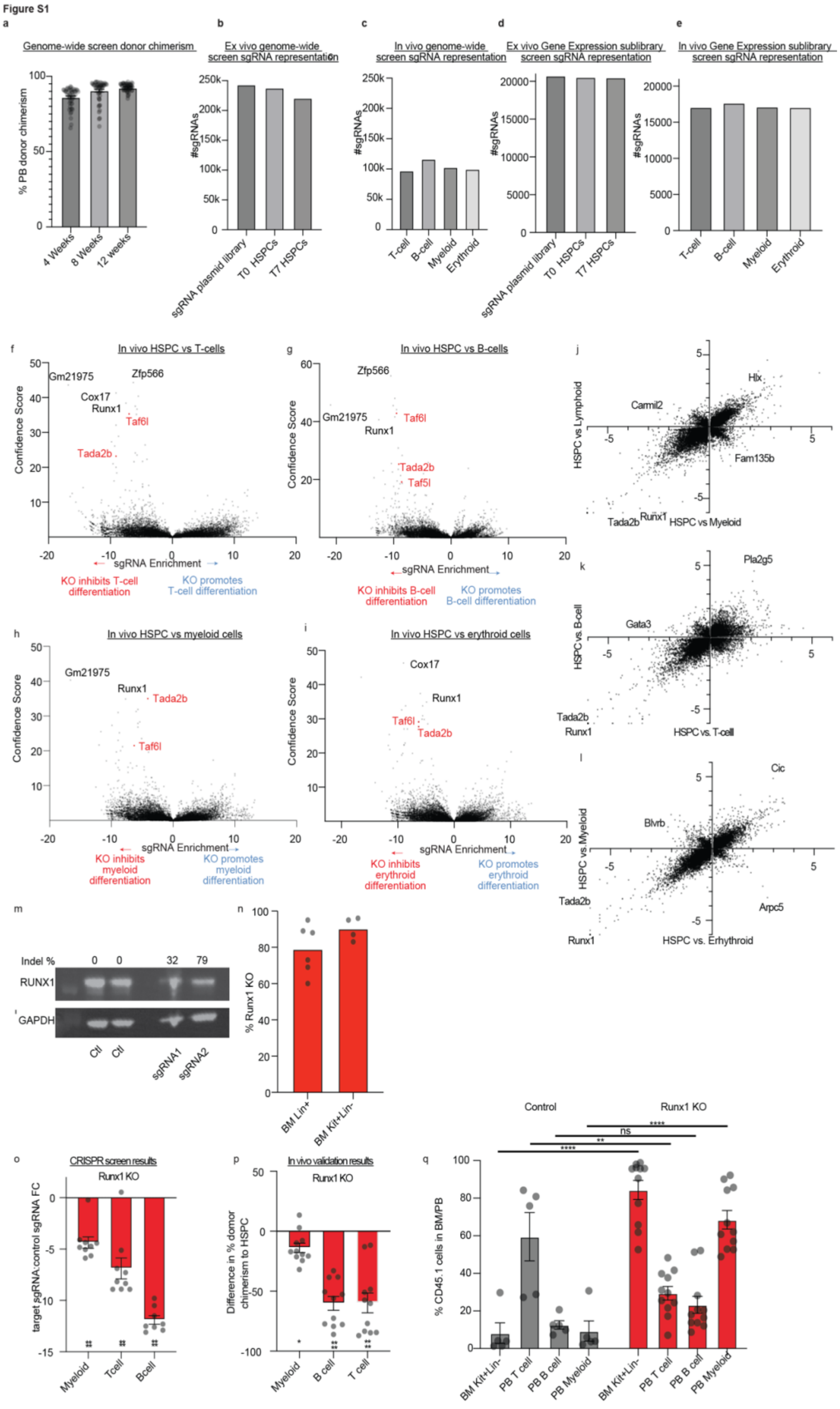

Figure S1: *In vivo* GW and GE-CRISPR KO screens

(a) Peripheral blood chimerism of GW CRISPR library cells across recipient mice.

(b) sgRNA library detection *ex vivo* for GW CRISPR screens (pre-transplantation).

(c) sgRNA library detection *in vivo* for GW CRISPR screens.

(d) sgRNA library detection *ex vivo* for gene expression sub-library CRISPR screens (pre-transplantation).

(e) sgRNA library detection *in vivo* for gene expression sub-library CRISPR screens.

(f-i) Volcano plot showing *in vivo* HSC CRISPR screen results comparing c-Kit$^+$Lineage$^-$ HSPC KOs with KO T-cells (b), B-cells (c), myeloid cells (d), and erythroid cells (e). All gene KOs displayed the effect of KO (negative if KO enriched in HSPCs, positive if KO enriched in differentiated cells) on the x axis and confidence score on the y axis. Saga Complex members labeled in red.

(j-l) Scatter plot of GW screens of Log(P-value) multiplied by sign of the effect score comparing HSCs and lymphoid cells with HSCs and myeloid cells (j), HSCs and B-cells with HSC and T-cells (k), and HSC and myeloid cells with HSCs and erythroid cells (k). Data can be accessed through webapp: www.hematopoiesiscrisprscreens.com

(m) Western blot for RUNX1 and GAPDH expression in control and *Runx1* RNP KO targeted HSCs. Percentage INDELS for each sample indicated above.

(n) *Runx1* KO frequencies in indicated bone marrow populations in primary recipients at 12-weeks after transplantation.

(o) Fold-change in *Runx1*-targeting sgRNA abundance in between the KSL HSPC compartment and myeloid, T and B cells, from GW screen results described.

(p) Difference in donor chimerism between the c-Kit$^+$Lineage$^-$ HSPC compartment and myeloid, T and B cells for *Runx1* KO HSPCs 12-weeks after transplantation. 10,000 CD45.1$^+$ *Runx1* KO HSCs were transplanted against 1 million CD45.1$^+$CD45.2$^+$ whole bone marrow competitor cells into lethally irradiated CD45.2$^+$ recipient mice.

(q) Donor chimerism from control and *Runx1* KO HSPCs 12-weeks after transplantation. 10,000 CD45.1$^+$ *Runx1* KO HSCs were transplanted against 1 million CD45.1$^+$CD45.2$^+$ whole bone marrow competitor cells into lethally irradiated CD45.2$^+$ recipient mice. Error bars represent s.e.m; P-value determined by two-way ANOVA.

Figure S2

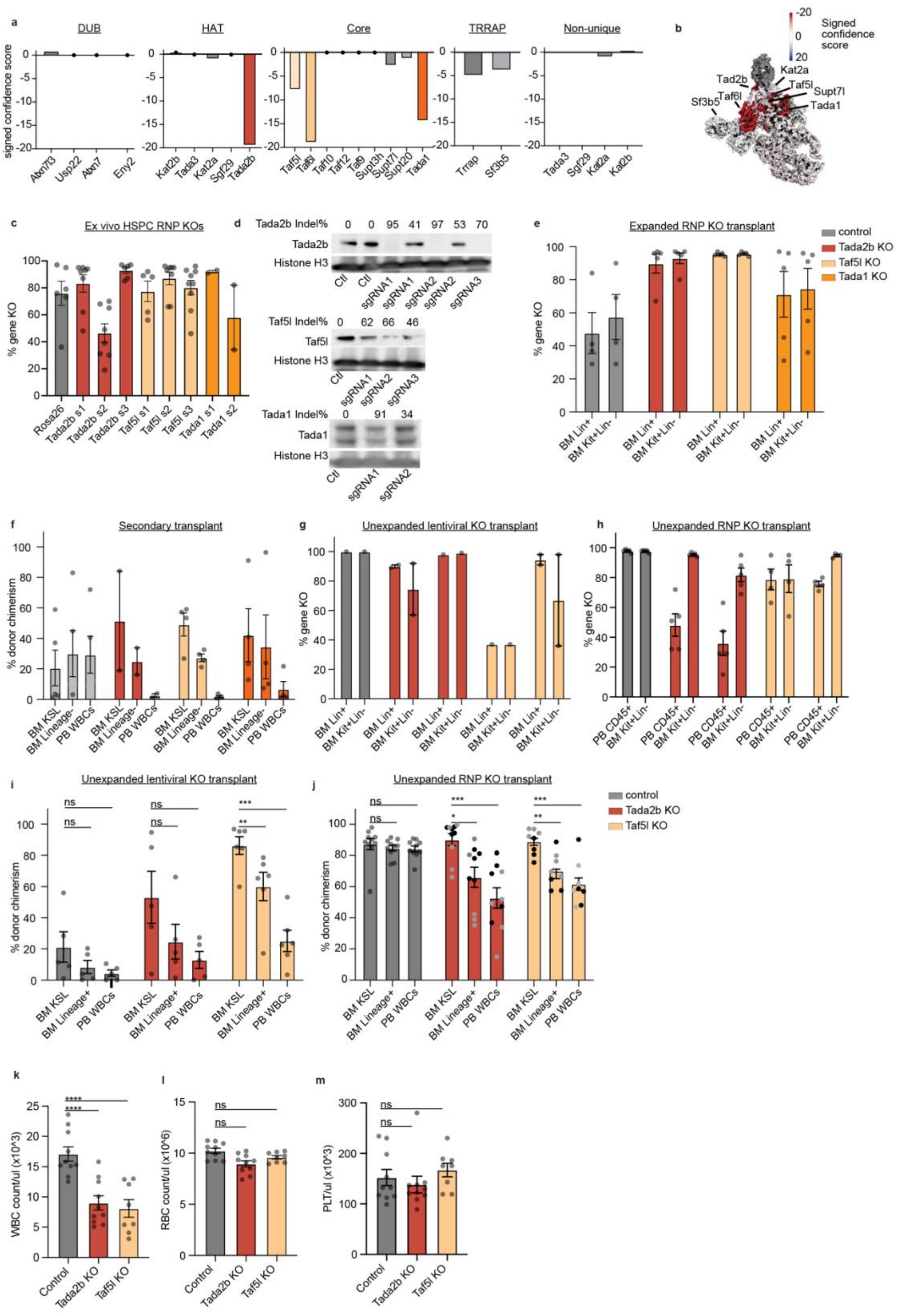

Figure S2: Analysis of SAGA component KO HSCs

(a) Signed confidence scores of GW screen data of members of SAGA complex and ATAC complex divided by structural subunits.

(b) 3D model of SAGA complex colored by signed confidence score values from GW screen data. Figure made using PyMol 3.1.

(c) *Ex vivo* HSPC gene KO frequencies with sgRNAs used in this study.

(d) Western blots showing TADA2B, TAF5L, and TADA1 protein levels in KO cells. Percentage INDELS for each sample indicated above.

(e) Gene KO frequencies in indicated bone marrow cell populations 12-weeks after transplantation described in Figure 2a.

(f) Donor chimerism within bone marrow and peripheral blood within secondary recipients at 16-weeks post-transplantation for transplants described in Figure 2a.

(g) Gene KO frequencies in indicated bone marrow populations in primary recipients 16-weeks post-transplantation of freshly-isolated $CD150^{+}CD34^{-}c\text{-}Kit^{+}Sca\text{-}1^{+}Lineage^{-}$ HSCs using lentiviral sgRNAs in Cas9 expressing HSPCs.

(h) Gene KO frequencies in indicated bone marrow populations in primary recipients 16-weeks post-transplantation of freshly-isolated $CD150^{+}CD34^{-}c\text{-}Kit^{+}Sca\text{-}1^{+}Lineage^{-}$ HSCs using RNP KO approaches.

(i) Donor chimerism within the bone marrow and peripheral blood from freshly-isolated $CD150^{+}CD34^{-}c\text{-}Kit^{+}Sca\text{-}1^{+}Lineage^{-}$ HSC KOs at 16-weeks post-transplantation using lentiviral sgRNAs in Cas9 expressing HSPCs. n=5 independent replicates. P-value determined by one-way ANOVA. Error bars represent s.e.m. ***P<.0005, **P<.005.

(j)Donor chimerism within the bone marrow and peripheral blood from freshly-isolated $CD150^{+}CD34^{-}c\text{-}Kit^{+}Sca\text{-}1^{+}Lineage^{-}$ HSC KOs at 16-weeks post-transplantation using RNP KO approaches. sgRNA 1 represented in black dots and sgRNA 2 represented in grey dots. P-value determined by one-way ANOVA. Error bars represent s.e.m. ***P<.0005, **P<.005.

(k) White blood cell (WBC) counts in transplant recipients from (j) at 16-weeks. Error bars represent s.e.m; P-value determined by one-way ANOVA.

(l) Red blood cell (RBC) counts in transplant recipients from (j) at 16-weeks. Error bars represent s.e.m; P-value determined by one-way ANOVA.

(m) Platelet (Plt) counts in transplant recipients from (j) at 16-weeks. Error bars represent s.e.m; P-value determined by one-way ANOVA.

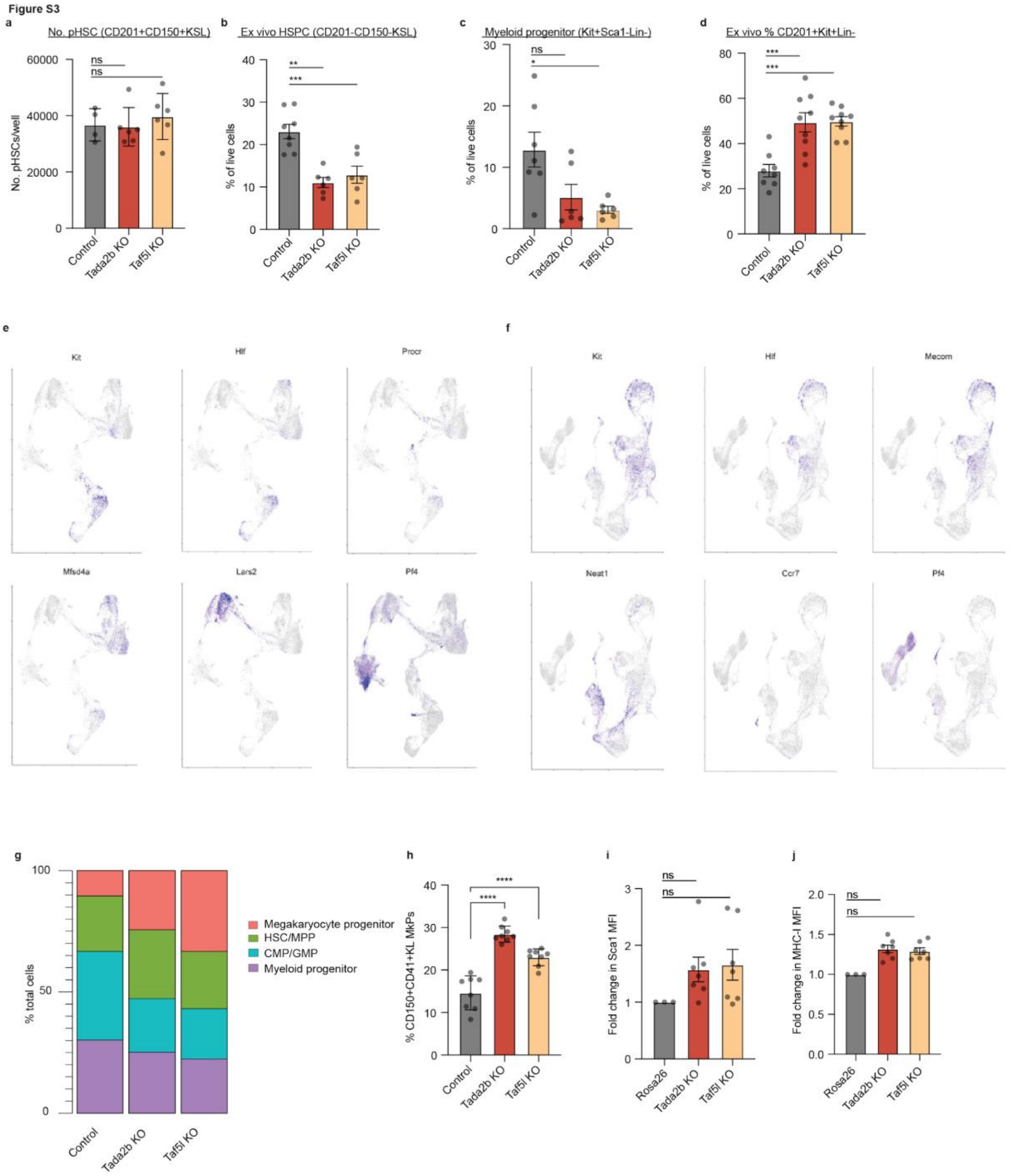

Figure S3: Analysis of SAGA component KO HSCs *ex vivo*

(a) Number of immunophenotypic HSCs (CD201$^+$CD150$^+$KSL) per well for each given condition. n=6 independent samples. Error bars represent s.e.m; P-value determined by one-way ANOVA.

(b) Percentage of CD201$^-$CD150$^-$KSL progenitor cells in *ex vivo* cultures 14-days after RNP KO of SAGA complex members. Six biological replicates with two independent sgRNAs displayed. n=6 independent samples. Error bars represent s.e.m; P-value determined by one-way ANOVA.

(c) Percentage of c-Kit$^+$Sca-1$^-$Lineage$^-$ progenitor cells in *ex vivo* cultures 14-days after RNP KO of SAGA complex members. Two independent sgRNAs displayed. Error bars represent s.e.m; n=6 independent samples. P-value determined by one-way ANOVA.

(d) Percentage of CD201$^+$c-Kit$^+$Lin$^-$ cells in *ex vivo* cultures 14-days after RNP KO of SAGA complex members. Two independent sgRNAs displayed. n=6 independent samples. Error bars represent s.e.m; P-value determined by one-way ANOVA.

(e) Feature plots for genes defining clusters in entire single cell dataset.

(f) Feature plots for genes defining clusters within the HSC cluster.

(g) Frequency of cell type clusters in the control, *Tada2b* KO, and *Taf5l* KO cultures as determined by single cell RNA-seq data.

(h) Frequency of MkPs (CD150$^+$CD41$^+$KL) within *ex vivo* HSPC cultures 14-days after RNP KO of SAGA complex members. n=8 biological replicates with two independent sgRNAs displayed. Error bars represent s.e.m; P-value determined by one-way ANOVA.

(i) Fold-change in flow cytometric Sca-1 MFI within *ex vivo* HSPC cultures 14-days after RNP KO of SAGA complex members. n=6 biological replicates with two independent sgRNAs displayed. Error bars represent s.e.m; P-value determined by one-way ANOVA.

(j) Fold-change in flow cytometric MHC-I MFI within *ex vivo* HSPC cultures 14-days after RNP KO of SAGA complex members. n=6 biological replicates with two independent sgRNAs displayed. Error bars represent s.e.m; P-value determined by one-way ANOVA.

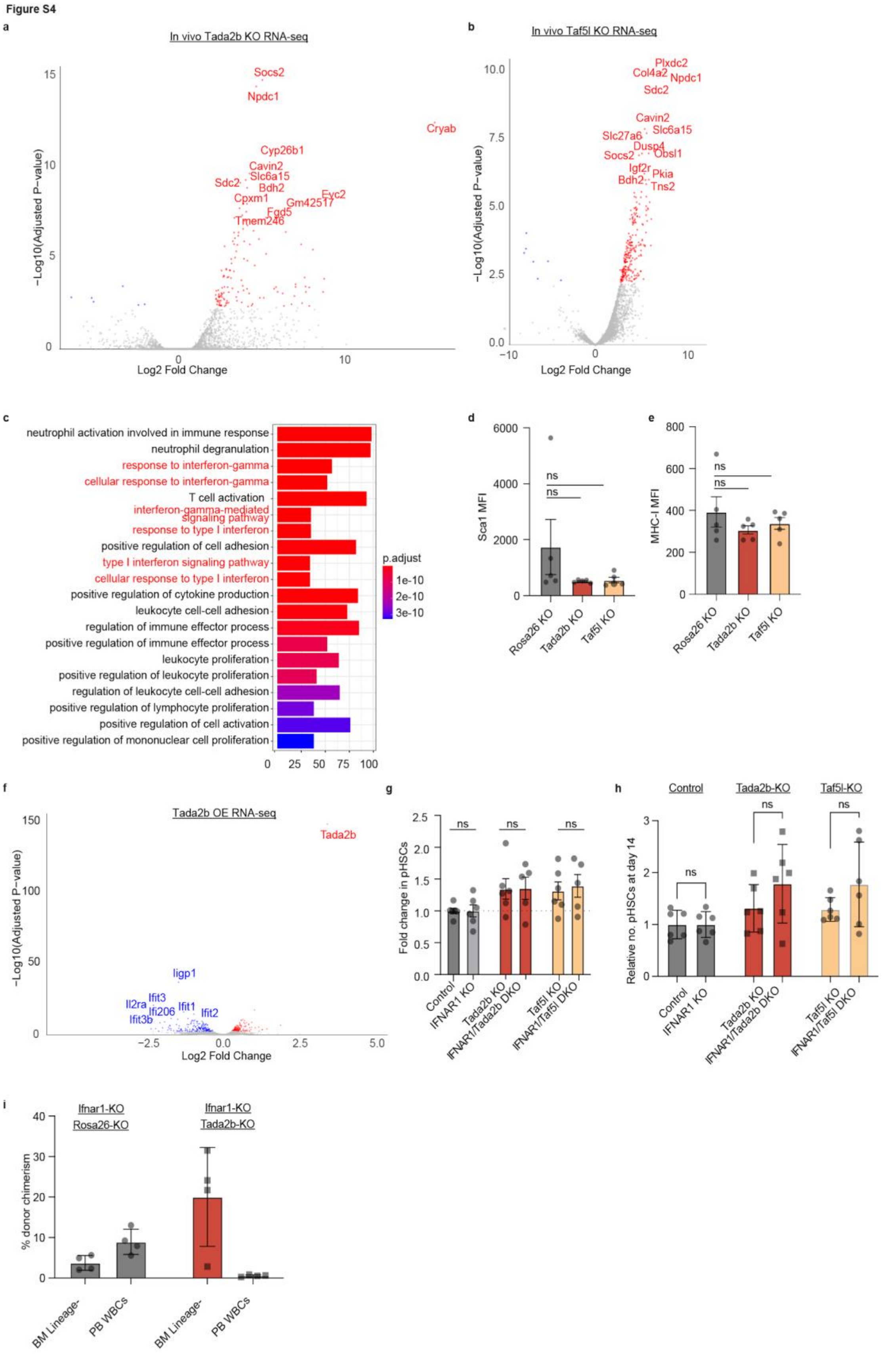

Figure S4: HSC transcriptional consequences of perturbing SAGA complex members

(a) Volcano plot comparing gene expression between *in vivo* control KSL HSPC with *in vivo* *Tada2b* KO KSL HSPCs, isolated at 16-weeks post-transplantation from recipient mice that received unexpanded RNP KO HSPCs.

(b) Volcano plot comparing gene expression between *in vivo* control KSL HSPC with *in vivo* *Taf5l* KO KSL HSPCs, isolated at 16-weeks post-transplantation from recipient mice mice that received unexpanded RNP KO HSPCs.

(c) GO enrichment of top 200 upregulated genes in *Tada2b* KO KSL HSPCs *in vivo* compared with control *in vivo* KSL HSPCs isolated at 16-weeks post-transplantation from recipient mice.

(d) Sca-1 MFI within donor c-Kit$^+$Lineage$^-$ bone marrow cells at 12-weeks post transplantation in recipient mice.

(e) MHC-I MFI within donor c-Kit$^+$Lineage$^-$ bone marrow cells at 12-weeks post transplantation in recipient mice.

(f) Volcano plot comparing gene expression between GFP-transduced KSL HSCs and *Tada2b*-transduced KSL HSCs, at 3-days culture post-transduction.

(g) Fold change in phenotypic mouse HSCs (CD201$^+$CD150$^+$KSL) frequency within the indicated HSPC cultures. n=6 biological replicates with two independent sgRNAs displayed. Error bars represent s.e.m; P-value determined by one-way ANOVA.

(h) Relative number of pHSCs (CD201$^+$CD150$^+$KSL) within the indicated HSPC cultures. n=6 biological replicates with two independent sgRNAs displayed. Error bars represent s.e.m; P-value determined by one-way ANOVA.

(i) Percent bone marrow chimerism from denoted cells KO cells in bone marrow and peripheral blood. n=4 biological replicates with two independent sgRNAs displayed. Error bars represent s.e.m.

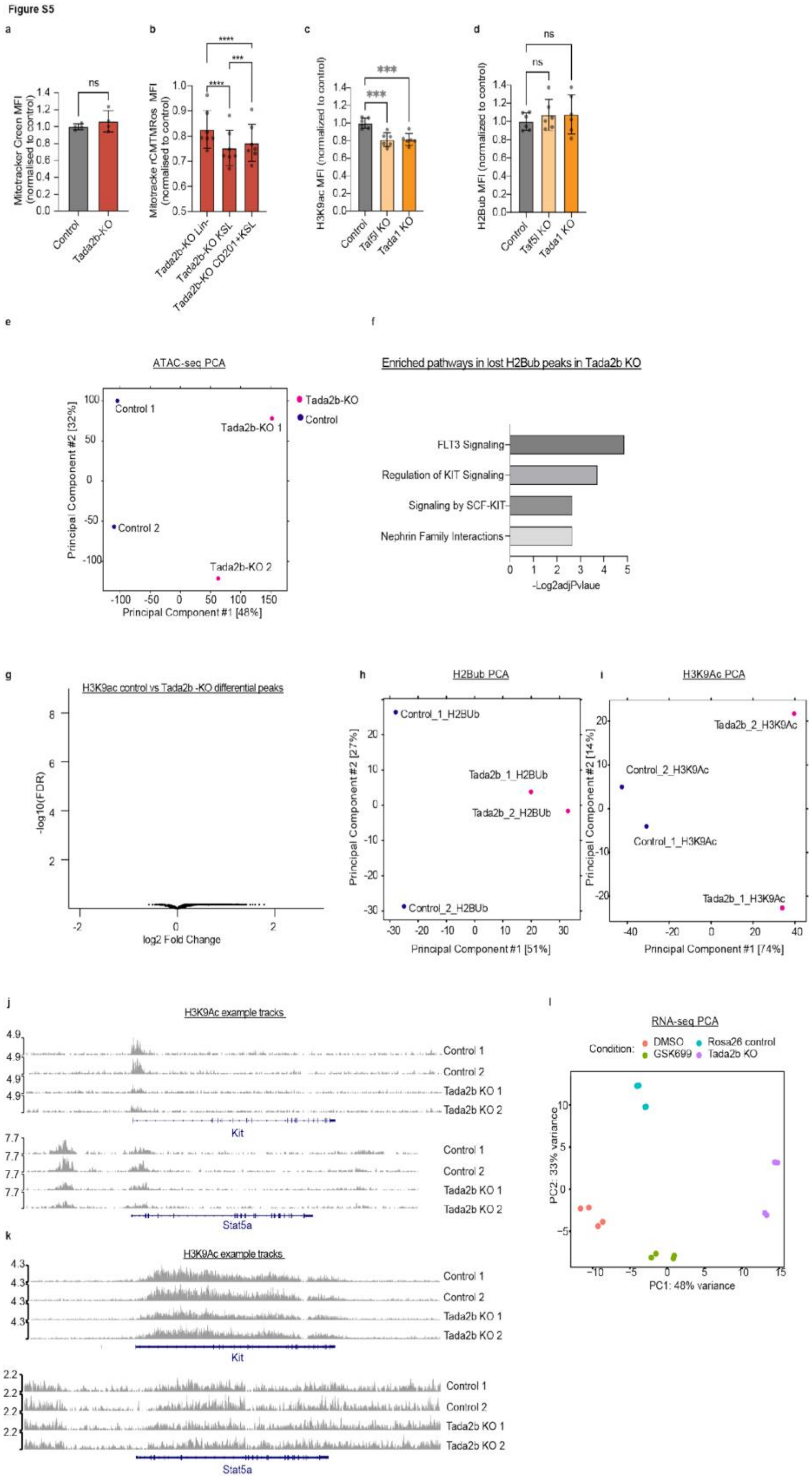

Figure S5: Epigenetic consequences of perturbing SAGA complex members in HSPCs

(a) Flow cytometric MitoTracker Green FM MFI in indicated HSPCs. n=4 biological replicates. Error bars represent s.e.m; P-value determined by unpaired T-test.

(b) Flow cytometric MitoTracker Orange CMTROS MFI in indicated HSPCs. n=6 biological replicates. Error bars represent s.e.m; P-value determined by one-way ANOVA.

(c) Intracellular flow cytometry for H3K9ac in indicated HSPCs. n=6 independent replicates. Error bars represent s.e.m; P-value determined by one-way ANOVA. ***P<.0005.

(d) Intracellular flow cytometry for H2Bub in indicated HSPCs. n=6 independent replicates. Error bars represent s.e.m; P-value determined by one-way ANOVA.

(e) PCA plot representing samples from ATAC-seq data.

(f) Reactome analysis of genes annotated near differentially lost H2Bub peaks in Tada2b KO HSPCs compared to control HSPCs.

(g) Volcano plot of differential peak analysis of H3Kac Chipmentation sequencing of Tada2b KO HSPCs compared to control HSPCs.

(h) PCA plot representing samples from H2Bub Chipmentation data.

(i) PCA plot representing samples from H3Kac Chipmentation data.

(j) Example tracks produced from H3Kac Chipmentation data plotted using IGV.

(k) Example tracks produced from H2Bub Chipmentation data plotted using IGV.

(l) PCA plot representing samples from bulk RNA-seq data of GSK699 treated HSPCs along with control and *Tada2b*-KO HSPCs.

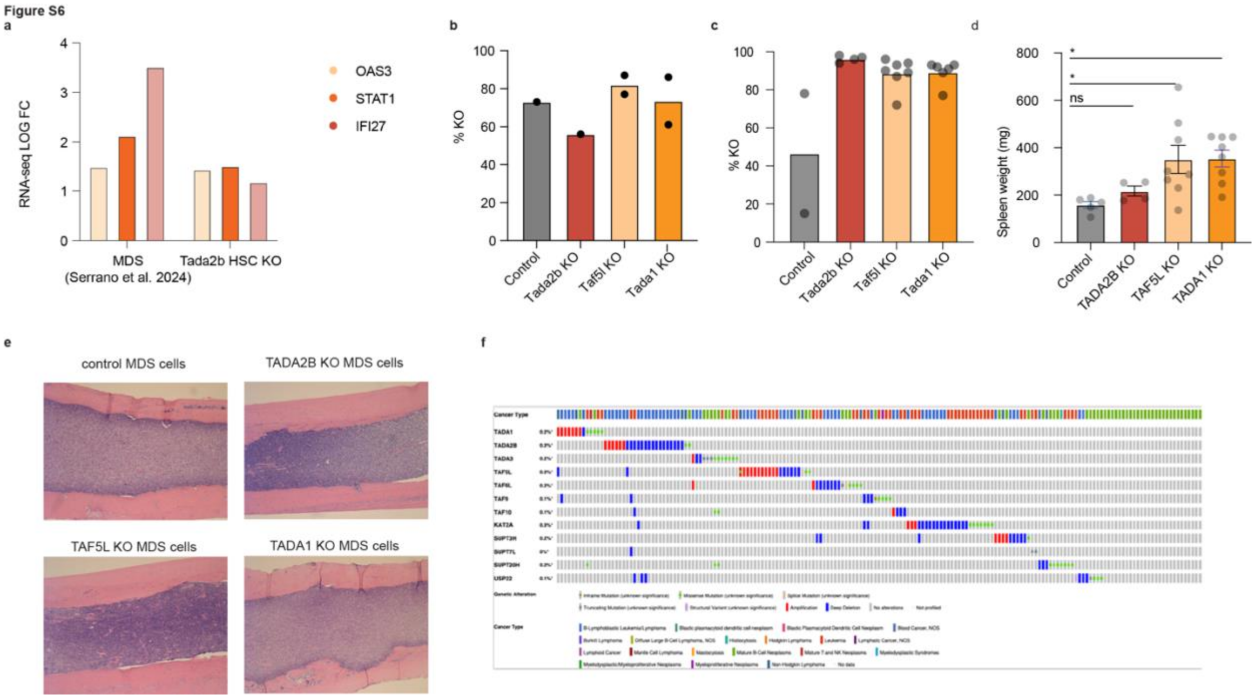

Figure S6: Role of SAGA complex members in human MDS

(a) Comparison of log fold change in gene expression in selected genes in human MDS samples (Serrano et al. 2024) compared with Tada2b HSPC KO cells.

(b) Gene KO frequencies in human MDS-L cells prior to transplantation. One *TADA2B* sgRNA failed to induce any mutation and was excluded from the transplant.

(c) Gene KO frequencies in MDS-L sgRNA-transduced cells at 12-weeks post-transplantation.

(d) Recipient mouse spleen weights at 12-week post-transplantation of control and KO MDS-L cells.

(e) Representative H&E-stained bone marrow sections from recipient mice at 12-week post-transplantation.

(f) Mutational frequency of SAGA complex members in blood cancers. Figure made using catalogue of somatic mutations in cancer (COSMIC).

Figure S7

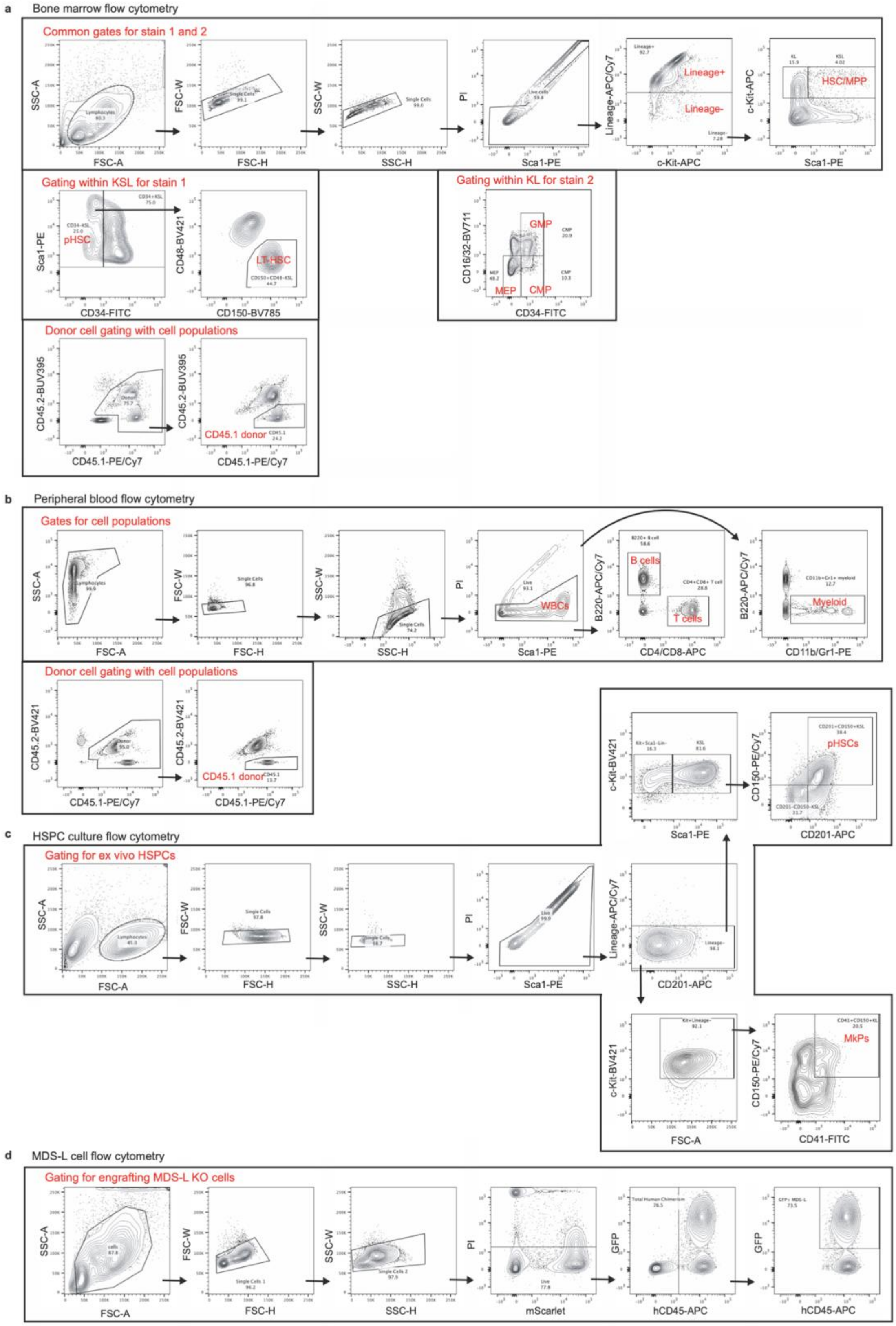

Figure S7: Representative flow cytometry gating

(a) Bone marrow flow cytometry gating strategy for mouse HSPC subsets and donor chimerism.

(b) Peripheral blood flow cytometry gating strategy for mouse immune cell types and donor chimerism.

(c) HSPC culture flow cytometry gating strategy for mouse HSPC cell types.

(d) Bone marrow flow cytometry gating strategy for human MDS-L cell chimerism.
