# Supplementary material for: In vivo CRISPR screening identifies SAGA complex members as key regulators of hematopoiesis
Source: Nat Commun. 2026 Jan 23;17:1756. doi: 10.1038/s41467-026-68465-6 (PMC12914052; doi:10.1038/s41467-026-68465-6)
Supplement: Supplementary file 2 — Description of Additional Supplementary Files [file 41467_2026_68465_MOESM2_ESM.pdf]

Description of Additional Supplementary Files

File Name: Supplementary Data 1
Description: **GW-CRISPR KO screen analysis results**
Analysis of GW-CRISPR screen using CasTLE. See excel spreadsheet.

File Name: Supplementary Data 2
Description: **GE-CRISPR KO screen analysis results**
Analysis of GE-CRISPR screen using CasTLE. See excel spreadsheet.

File Name: Supplementary Data 3
Description: **Single-cell RNA-seq from single-cell RNA-seq comparing WT vs *Tada2b* KO cells**
DEG analysis of single-cell RNA-seq data comparing *Tada2b* KO and WT cells separated by cell type. See excel spreadsheet.

File Name: Supplementary Data 4
Description: **Single-cell RNA-seq analysis results comparing WT vs *Taf5l* KO cells**
DEG analysis of single-cell RNA-seq data comparing *Taf5l* KO and WT cells separated by cell type. See excel spreadsheet.

File Name: Supplementary Data 5
Description: **Bulk RNA-seq analysis results comparing *in vivo* control vs *Taf5l* KO and *Tada2b* KO cells**
DEG analysis of bulk RNA-seq data comparing *Tada2b*-KO *and Taf5l*-KO *in vivo* HSPCs compared WT HSCs. See excel spreadsheet.

File Name: Supplementary Data 6
Description: **Bulk RNA-seq analysis results for *in vitro* experiments**
DEG analysis of bulk RNA-seq data comparing *Tada2b*-OE, *Tada2b-KO, Ifnar-KO, Ifnar-Tada2b-DBKO* and control HSPCs. See excel spreadsheet.

File Name: Supplementary Data 7
Description: **ATAC-Seq and Chipmentation data of *Tada2b* KO and Control HSPCs**
Differential Peak analysis, HOMER enrichment motif analysis and Gene enrichment terms for ATAC-seq data, and H2Bub and H3K9ac Chipmentation data comparing Tada2b KO and control HSPCs .

File Name: Supplementary Data 8
Description: **Bulk RNA-seq analysis results for HSPCs treated with KAT2A/B inhibitor (GSK699).**
DEG analysis of bulk RNA-seq data of HSPCs treated with KAT2A/B inhibitor (GSK699) and KO of *Tada2b* along untreated WT HSPCs and Rosa26 KO controls.

File Name: Supplementary Data 9
Description: **Bulk RNA-seq analysis results for *in vivo* experiments comparing *TADA2B*-KO, *TAF5L*-KO, *TADA1*-KO MDS-L cells compared to control MDS-L cells**
DEG analysis of bulk RNA-seq data comparing *TADA2B*-KO, *TAF5L*-KO, *TADA1*-KO MDS-L cells compared to control MDS-L cells.
