# Supplementary material for: In vivo CRISPR screening identifies SAGA complex members as key regulators of hematopoiesis
Source: Nat Commun. 2026 Jan 23;17:1756. doi: 10.1038/s41467-026-68465-6 (PMC12914052; doi:10.1038/s41467-026-68465-6)
Supplement: Supplementary file 12 — Reporting Summary [file 41467_2026_68465_MOESM12_ESM.pdf]

Corresponding author(s): Wilkinson

Last updated by author(s): Dec 23, 2025

Reporting Summary

Nature Portfolio wishes to improve the reproducibility of the work that we publish. This form provides structure for consistency and transparency in reporting. For further information on Nature Portfolio policies, see our Editorial Policies and the Editorial Policy Checklist.

Statistics

For all statistical analyses, confirm that the following items are present in the figure legend, table legend, main text, or Methods section.

n/a	Confirmed	
☐	☒	The exact sample size (*n*) for each experimental group/condition, given as a discrete number and unit of measurement
☐	☒	A statement on whether measurements were taken from distinct samples or whether the same sample was measured repeatedly
☐	☒	The statistical test(s) used AND whether they are one- or two-sided<br>*Only common tests should be described solely by name; describe more complex techniques in the Methods section.*
☐	☒	A description of all covariates tested
☐	☒	A description of any assumptions or corrections, such as tests of normality and adjustment for multiple comparisons
☐	☒	A full description of the statistical parameters including central tendency (e.g. means) or other basic estimates (e.g. regression coefficient) AND variation (e.g. standard deviation) or associated estimates of uncertainty (e.g. confidence intervals)
☐	☒	For null hypothesis testing, the test statistic (e.g. *F*, *t*, *r*) with confidence intervals, effect sizes, degrees of freedom and *P* value noted<br>*Give P values as exact values whenever suitable.*
☒	☐	For Bayesian analysis, information on the choice of priors and Markov chain Monte Carlo settings
☒	☐	For hierarchical and complex designs, identification of the appropriate level for tests and full reporting of outcomes
☒	☐	Estimates of effect sizes (e.g. Cohen's *d*, Pearson's *r*), indicating how they were calculated

Our web collection on statistics for biologists contains articles on many of the points above.

Software and code

Policy information about availability of computer code

Data collection	FACSDiva
Data analysis	GraphPad Prism 10.1.1, FlowJo 10.8.0, CasTLE 1.0, DESeq2 1.42.1, Seurat 2.0

For manuscripts utilizing custom algorithms or software that are central to the research but not yet described in published literature, software must be made available to editors and reviewers. We strongly encourage code deposition in a community repository (e.g. GitHub). See the Nature Portfolio guidelines for submitting code & software for further information.

Data

Policy information about availability of data

All manuscripts must include a data availability statement. This statement should provide the following information, where applicable:

- Accession codes, unique identifiers, or web links for publicly available datasets
- A description of any restrictions on data availability
- For clinical datasets or third party data, please ensure that the statement adheres to our policy

Data supporting the findings of this work are available within the paper and its Supplementary Raw datasets and materials generated and analyzed during the current study are available via NCBI's Gene Expression Omnibus (GEO) under accession numbers GSE312143 [https://www.ncbi.nlm.nih.gov/geo/query/acc.cgi?acc=GSE312143] (CRISPR screen), GSE312125 [https://www.ncbi.nlm.nih.gov/geo/query/acc.cgi?acc=GSE312125] (scRNA-seq), GSE312282 [https://www.ncbi.nlm.nih.gov/geo/query/acc.cgi?acc=GSE312282] (bulk RNA-seq), GSE312290 [https://www.ncbi.nlm.nih.gov/geo/query/acc.cgi?acc=GSE312290] (ATAC-

seq), GSE312126 [https://www.ncbi.nlm.nih.gov/geo/query/acc.cgi?acc=GSE312126] (ChIP-seq). The genome-wide CRISPR screen data are available via an interactive web application: www.hematopoiesiscrisprscreens.com.

Research involving human participants, their data, or biological material

Policy information about studies with human participants or human data. See also policy information about sex, gender (identity/presentation), and sexual orientation and race, ethnicity and racism.

Reporting on sex and gender	Fully anonymised umbilical cord blood was provided by the NHSBT. No sex information available.
Reporting on race, ethnicity, or other socially relevant groupings	Fully anonymised umbilical cord blood was provided by the NHSBT. No sex information available.
Population characteristics	N/A
Recruitment	N/A
Ethics oversight	Human primary cell experiments were approved by the University of Oxford's research ethics committee (OxTREC-574-23)

Note that full information on the approval of the study protocol must also be provided in the manuscript.

Field-specific reporting

Please select the one below that is the best fit for your research. If you are not sure, read the appropriate sections before making your selection.

☒ Life sciences ☐ Behavioural & social sciences ☐ Ecological, evolutionary & environmental sciences

For a reference copy of the document with all sections, see nature.com/documents/nr-reporting-summary-flat.pdf

Life sciences study design

All studies must disclose on these points even when the disclosure is negative.

Sample size	No statistical methods were used to predetermine the experimental sample size.
Data exclusions	No data were excluded
Replication	For all figures, multiple independent experiments were performed and all attempts at replicating observation as described in the manuscript were successful.
Randomization	For transplantation assays, mice were randomly allocated between groups
Blinding	For transplantation assays, injections were performed blinded.

Reporting for specific materials, systems and methods

We require information from authors about some types of materials, experimental systems and methods used in many studies. Here, indicate whether each material, system or method listed is relevant to your study. If you are not sure if a list item applies to your research, read the appropriate section before selecting a response.

Materials & experimental systems

n/a	Involved in the study	
☐	☒	Antibodies
☐	☒	Eukaryotic cell lines
☒	☐	Palaeontology and archaeology
☐	☒	Animals and other organisms
☒	☐	Clinical data
☒	☐	Dual use research of concern
☒	☐	Plants

Methods

n/a	Involved in the study	
☐	☒	ChIP-seq
☐	☒	Flow cytometry
☒	☐	MRI-based neuroimaging

Antibodies

Antibodies used	Alexa Fluor 700 anti-mouse CD34 (Thermo Fisher, cat. #56-0341-82), Alexa Fluor 700 anti-mouse H-2Kb (MHC class I) (BioLegend, cat.

Antibodies used	#116522), anti-acetyl-Histone H3 (Lys9) (Merck Millipore, cat. #07-352), anti-mouse GAPDH (Bethyl Laboratories, cat. #A300-641A), anti-mouse Histone H3 (Abcam, cat. #ab1791), anti-mouse RUNX1 (Cell Signaling Technology, cat. #8529s), anti-mouse TADA1 (Proteintech, cat. #20337-1-AP), anti-mouse TADA2B (St. John's Laboratory, cat. #STJ194361-200), anti-mouse TAF5L (Proteintech, cat. #19274-AP), anti-ubiquityl-Histone H2B (Lys120) (Cell Signaling Technology, cat. #5546), APC anti-human CD41 (BioLegend, cat. #303710), APC anti-mouse CD4 (BioLegend, cat. #100516), APC anti-mouse CD8a (BioLegend, cat. #100712), APC anti-mouse CD117 (c-Kit) (BioLegend, cat. #105812), APC anti-mouse CD201 (EPCR) (Thermo Fisher, cat. #17-2012-82), APC anti-mouse Ly-6G/Ly-6C (Gr-1) (BioLegend, cat. #108412), APC anti-mouse Ter119 (BioLegend, cat. #116212), APC anti-mouse/human CD11b (BioLegend, cat. #101212), APC anti-mouse/human CD45R (BioLegend, cat. #103212), APC/Cy7 anti-human CD34 (BioLegend, cat. #343514), APC/Cy7 anti-mouse CD41 (BioLegend, cat. #133927), APC/Cyanine7 anti-mouse/human CD45R/B220 (BioLegend, cat. #103224), APC/Cyanine7 Streptavidin (BioLegend, cat. #405208), Biotin anti-mouse CD4 (BioLegend, cat. #100508), Biotin anti-mouse CD8a (BioLegend, cat. #100704), Biotin anti-mouse CD127 (IL-7Rα) (BioLegend, cat. #135006), Biotin anti-mouse Ly-6G/Ly-6C (Gr-1) (BioLegend, cat. #108404), Biotin anti-mouse TER-119/Erythroid Cells (BioLegend, cat. #116204), Biotin anti-mouse/human CD45R/B220 (BioLegend, cat. #103204), Brilliant Violet 421 anti-mouse CD48 (BioLegend, cat. #103428), Brilliant Violet 421 anti-mouse CD117 (c-Kit) (BioLegend, cat. #105828), Brilliant Violet 711 anti-mouse CD16/32 (Fcγ R III/II) (BioLegend, cat. #101337), Brilliant Violet 785 anti-mouse CD150 (SLAM) (BioLegend, cat. #115937), BUV395 Mouse Anti-Mouse CD45.2 (BD Biosciences, cat. #564616), BV785 anti-human CD45RA (BioLegend, cat. #304140), eFluor 450 anti-mouse CD45.2 (Thermo Fisher, cat. #48-0454-82), FITC anti-mouse CD4 (BioLegend, cat. #100510), FITC anti-mouse CD8a (BioLegend, cat. #100706), FITC anti-mouse CD34 (Thermo Fisher, cat. #11-0341-85), FITC anti-mouse CD41 (BioLegend, cat. #133903), FITC anti-mouse CD127 (IL-7Rα) (BioLegend, cat. #135008), FITC anti-mouse Ly-6G/Ly-6C (Gr-1) (BioLegend, cat. #108406), FITC anti-mouse Ter119 (BioLegend, cat. #116206), FITC anti-mouse/human CD45R/B220 (BioLegend, cat. #103206), Microbead-conjugated anti-APC (Miltenyi Biotec, cat. #130-090-855), Microbead-conjugated anti-mouse CD117 (c-Kit) (Miltenyi Biotec, cat. #130-097-146), PE anti-acetyl-Histone H3 (Lys9) (Cell Signaling Technology, cat. #28036), PE anti-human CD201 (BioLegend, cat. #351904), PE anti-mouse Ly-6A/E (Sca-1) (BioLegend, cat. #108108), PE anti-mouse Ly-6G/Ly-6C (Gr-1) (BioLegend, cat. #108408), PE anti-mouse/human CD11b (BioLegend, cat. #101208), PE anti-ubiquityl-Histone H2B (Lys120) (Cell Signaling Technology, cat. #86653), PE/Cyanine7 anti-mouse CD45.1 (BioLegend, cat. #110730), PE/Cyanine7 anti-mouse CD150 (SLAM) (BioLegend, cat. #115914), Peroxidase conjugated goat anti-rabbit IgG (Sigma Aldrich, cat. #A6667). See Methods section for antibody dilution information.
Validation	All antibodies were validated by manufacturers for the applications and species used in this study. See manufacturers websites for validation statements (www.biolegend.com; www.thermofisher.com/us/en/home/life-science/antibodies/ebioscience ; www.bdbiosciences.com; www.cellsignal.com).

Eukaryotic cell lines

Policy information about cell lines and Sex and Gender in Research

Cell line source(s)	MDS-L cells: a kind gift from Tohyama laboratory, Kawasaki Medical School; NIH/3T3 cell line: ATCC CRL-1658
Authentication	Cell lines used were not authenticated
Mycoplasma contamination	Cell lines were not tested for mycoplasma contamination
Commonly misidentified lines (See ICLAC register)	N/A

Animals and other research organisms

Policy information about studies involving animals; ARRIVE guidelines recommended for reporting animal research, and Sex and Gender in Research

Laboratory animals	Mouse strains:<br>B6J.129(Cg)-Gt(ROSA)26Sortm1.1(CAG-cas9*,-EGFP)Fezh/J (JAX 026179)<br>C57BL/6-CD45.1 PepboyJ (JAX 002014)<br>C57BL/6-CD45.2 (JAX 000664)<br>NOD.Cg-Prkdcscid Il2rgtm1Sug Tg(SRa-IL3, CSF2)7-2/Jic (n-Vivo Science Inc. NOG-EXL)<br>B6.bkg-Ifnar1tm1Agt/Mmjax (provided by Caetano Reis e Sousa)<br><br>All mice were 8-12 weeks at the start of experiments<br>Both male and female mice were used.
Wild animals	This study did not involve wild animals.
Reporting on sex	Both sexes were used in experimental studies.
Field-collected samples	This study did not involve field-collected samples.
Ethics oversight	All animal experiments were approved by the Administrative Panel on Laboratory Animal Care at Stanford University, the UK Home Office, or the Animal Care and Use Committee of the Institute of Medical Science University of Tokyo.

Note that full information on the approval of the study protocol must also be provided in the manuscript.

Plants

Seed stocks	N/A
Novel plant genotypes	N/A
Authentication	N/A

ChIP-seq

Data deposition

☒ Confirm that both raw and final processed data have been deposited in a public database such as GEO.

☒ Confirm that you have deposited or provided access to graph files (e.g. BED files) for the called peaks.

Data access links<br>*May remain private before publication.*	GEO accession number: GSE312126
Files in database submission	2x control HSC H2Bub chipmentation<br>2x Tada2-KO HSC H2Bub chipmentation<br>2x control HSC H3K9ac chipmentation<br>2x Tada2b-KO HSC H3K9ac chipmentation
Genome browser session<br>(e.g. UCSC)	See Figure S5j-k for tracks.

Methodology

Replicates	Two biological replicates, both included in analysis
Sequencing depth	>20M reads per sample
Antibodies	Anti-acetyl histone H3 Lys9, Merck Millipore 07-352<br>Anti-ubiquityl histone H2B Lys120, Cell Signaling 5546
Peak calling parameters	Raw reads were assessed for quality using FASTQC and adapters were trimmed with Trimmomatic. Reads were mapped to the mm10 genome using Bowtie2, and SAM files were converted to BAM format with Samtools. The BAM files were sorted and indexed using Samtools, and mapping statistics were generated. Genome coverage was calculated using bedtools genomecov to produce bedGraph files. BigWig files were generated using bedGraphToBigWig.
Data quality	PCR duplicates were removed with Samtools, and BED files were created using bedtools bamtobed. Reads mapping to chrM and regions in the ENCODE mm10 blacklist were removed from the BED files.
Software	Differential peak analysis was performed using DiffBind, and differential peaks were annotated and analyzed for motif enrichment using HOMER.

Flow Cytometry

Plots

Confirm that:

☒ The axis labels state the marker and fluorochrome used (e.g. CD4-FITC).

☒ The axis scales are clearly visible. Include numbers along axes only for bottom left plot of group (a 'group' is an analysis of identical markers).

☒ All plots are contour plots with outliers or pseudocolor plots.

☒ A numerical value for number of cells or percentage (with statistics) is provided.

Methodology

Sample preparation	Bone marrow, peripheral blood, and in vitro cell cultures were prepared into a single cell suspension in PBS. Bone marrow

Sample preparation	cells were cKit-enriched using an MACS LS column (Miltenyi). Red blood cell lysis using aqueous 140 mM ammonium chloride was performed on peripheral blood cells. Cells were filters (40uM) before FACS.
Instrument	BD FACS AriaII, BS FACS Fusion, BD LSRFortessa, BD FACS Canto.
Software	FACS Diva for data collection, FlowJo for data analysis.
Cell population abundance	FACS machine cell sorting efficiency was confirmed by flow cytometric analysis of post-sorted cells.
Gating strategy	HSCs: FSC-A/SSC-A for mononuclear cells, FSC-H/FSC-W followed by SSC-H/SSC-W for singlets, PI/Sca1 for PI- live cells, Lineage-cocktail/cKit for Lin- cells, Sca1/CD34 for CD34-/lo Lin-, cKit/Sca1 for CD34-/lo cKit+ Sca1+ Lin-, CD150/blank channel for CD150+ CD34-/lo Kit+ Sca1+ Lin-. For peripheral blood analysis, live cells were gated based on positive and negative markers. For HSPC cultures, love cells were gated based on positive and negative markers.

☒ Tick this box to confirm that a figure exemplifying the gating strategy is provided in the Supplementary Information.
